# Oral health status and associated influencing factors in children with attention-deficit/hyperactivity disorder: a case-control study

**DOI:** 10.1186/s12903-026-09605-8

**Published:** 2026-08-19

**Authors:** Rui Dong, Han Wu, Feifei Jin, Qiqi Yang, Tongzhuo Chu, Hui Song

**Affiliations:** 1 Department of Stomatology, Dezhou Maternal and Child Health Hospital, Dezhou, Shandong China; 2 Department of Child Rehabilitation, Dezhou Maternal and Child Health Hospital, Dezhou, Shandong China; 3 Department of VIP Clinics, School and Hospital of Stomatology, Cheeloo College of Medicine, Shandong University & Shandong Key Laboratory of Oral Diseases & Shandong Engineering Research Center of Dental Materials and Oral Tissue Regeneration & Shandong Provincial Clinical Research Center for Oral Diseases, Jinan, 250012 China

**Keywords:** Attention-deficit/hyperactivity disorder, Oral health, Dental caries, Periodontal diseases, Children, Associated factors, Protective factors, Methylphenidate, Case-control study

## Abstract

**Background:**

Attention-deficit/hyperactivity disorder (ADHD) is the second most prevalent chronic pediatric condition worldwide. Its core ADHD symptoms—inattention, hyperactivity, and impulsivity—impair oral hygiene behaviors, and first-line medications such as methylphenidate may further increase oral disease risk. However, systematic epidemiological data on oral health status of children with ADHD remain limited in China. This study aimed to investigate oral health status of children with ADHD and identify factors associated with adverse oral health outcomes in this population.

**Methods:**

This case-control study included 130 children with ADHD and 143 age-matched healthy controls (6–12 years old) recruited from Dezhou Maternal and Child Health Hospital, Shandong Province, China, between July and December 2025. Oral examinations assessed dental caries (using the DMFT index for permanent teeth and dmft index for primary teeth, collectively reflecting the mixed-dentition caries burden), caries activity (Cariostat), periodontal status (Plaque Index [PI], Modified Gingival Index [MGI]), malocclusion, and dental trauma. Parent-reported questionnaires collected data on oral health behaviors, socioeconomic characteristics, and medication use. Between-group differences were analyzed using the Mann-Whitney U test, chi-square test, and Fisher exact test. Multivariate logistic regression with backward likelihood ratio selection was used to examine factors associated with adverse oral outcomes after adjusting for confounders. The English versions of the questionnaires are provided in Supplementary Files 1 and 2.

**Results:**

The ADHD group had significantly higher overall dental caries prevalence (80.77% vs. 52.45%, *P* < 0.001), higher median DMFT+dmft scores (3 vs. 1, *P* < 0.001), higher plaque index (PI) scores (2 vs. 1, *P* < 0.001), higher modified gingival index (MGI) scores (2 vs. 1, *P* < 0.001), and higher Cariostat values (*P* < 0.0001) than the control group. In contrast, the fissure sealant rate was significantly lower in the ADHD group (6.15% vs. 47.29%, *P* < 0.001). Multivariate analysis showed that methylphenidate use (adjusted odds ratios *[aOR]* = 39.65, *95% CI*: 15.79–271, *P* < 0.001) and frequent candy consumption (*aOR* = 84.54, *95% CI: 12.2*–584, *P* < 0.001) were associated with higher odds of dental caries in the adjusted model. In contrast, a higher parental education level (college degree or above) was associated with lower odds of dental caries (*aOR* = 0.10, *95% CI*: 0.014–0.684, *P* = 0.019). Perfunctory toothbrushing (*aOR* = 8.78, *95% CI*: 3.12–24.71, *P* < 0.001) was associated with higher odds of poor oral hygiene (PI ≥ 2), whereas higher household income (*aOR* = 0.29, *95% CI*: 0.12–0.74, *P* = 0.009) and twice-daily toothbrushing (*aOR* = 0.14, *95% CI*: 0.05–0.43, *P* = 0.001) were associated with lower odds of poor oral hygiene (PI ≥ 2). For gingivitis (MGI ≥ 2), higher household income (*aOR* = 0.33, *95% CI*: 0.13–0.84, *P* = 0.019) and parental supervision of toothbrushing (*aOR* = 0.11, *95% CI*: 0.04–0.31, *P* < 0.001) were associated with lower odds of gingivitis, whereas longer disease duration (*aOR* = 11.30, *95% CI*: 3.78–33.75, *P* < 0.001) was associated with higher odds of gingivitis. Notably, the wide confidence intervals for these adjusted odds ratios indicate limited precision for quantifying exact effect magnitudes, and the case-control design precludes causal inference; all reported associations should therefore be interpreted with caution.

Children with ADHD in this cohort exhibited significantly poorer oral health than healthy controls, characterized by higher rates of dental caries, poorer oral hygiene, and more severe gingival inflammation. These adverse oral health outcomes were associated with ineffective oral hygiene practices, frequent high-sugar dietary habits, ADHD-related neurobehavioral characteristics, and socioeconomic factors. Comprehensive oral health interventions integrating preventive clinical care, behavioral support, family involvement, and socioeconomic assistance are urgently needed for this population.

**Supplementary Information:**

The online version contains supplementary material available at https://doi.org/10.1186/s12903-026-09605-8.

## Background

Oral health is a critical component of overall health during childhood. Pediatric oral diseases are dominated by dental caries and periodontal diseases [1]. Dental caries is the most common chronic oral disorder in children and has a multifactorial pathogenesis involving oral microbial dysbiosis, dietary patterns, and oral hygiene behaviors [2]. According to the Fourth National Oral Health Epidemiological Survey, the prevalence of dental caries among Chinese children was 70.9% at 5 years of age and 34.5% at 12 years of age [3], highlighting the substantial burden of pediatric oral disease in China. Periodontal diseases in children are predominantly manifested as gingivitis, with gingival bleeding as a typical clinical sign [4]. Inadequately controlled gingivitis may progress to involve deeper periodontal structures, leading to adverse long-term oral health outcomes [5].

Attention-deficit/hyperactivity disorder (ADHD) is a highly prevalent neurodevelopmental disorder characterized by inattention, hyperactivity, and impulsivity [6]. It affects approximately 2%–7% of school-aged children globally [7], and is considered the most common chronic pediatric neurological condition—second only to asthma among all chronic childhood diseases, with an estimated global prevalence of approximately 5% [8]. A nationwide survey by Wang et al. reported an ADHD prevalence of approximately 6.3% among school-aged children in China [9], indicating that millions of children are affected and that ADHD imposes a significant burden on individuals, families, and society. Due to deficits in attention, executive functioning, and behavioral regulation, children with ADHD often experience difficulty maintaining effective daily oral hygiene practices [10]. Moreover, first-line pharmacotherapy for ADHD, particularly methylphenidate, is associated with oral adverse effects including xerostomia, bruxism, and taste alterations, which may further increase the risk of oral diseases [11].

International evidence consistently indicates that children with ADHD exhibit poorer oral health than their typically developing peers. Reported dental caries prevalence ranges from approximately 60% to 85% among children with ADHD, compared with 35%–55% in healthy controls [12–15]. In addition, plaque index and gingival index scores are consistently higher in children with ADHD [15]. However, existing literature yields heterogeneous results and has mainly focused on Western and Eastern European cohorts, where dietary patterns, oral healthcare systems, and ADHD management practices differ substantially from those in China. Therefore, the generalizability of these findings to Chinese children remains uncertain.

Although interest in the association between ADHD and oral health has increased globally, the available evidence is still limited and fragmented, and data from China are lacking [12–17]. To our knowledge, no systematic epidemiological survey has investigated the oral health status of children with ADHD in China. Given the potential differences in dietary habits, oral healthcare services, and ADHD management practices between China and other countries, findings from international studies may not be directly applicable to the Chinese population. This case-control study was therefore conducted to systematically investigate oral health status of children with ADHD and associated influencing factors in Dezhou, Shandong Province. The findings are expected to provide baseline evidence to support the development of targeted oral health promotion and intervention strategies for this underserved population.

## Methods

### Study subjects

The ADHD group included 130 children aged 6–12 years who were diagnosed with attention-deficit/hyperactivity disorder (ADHD) and attended the Department of Pediatric Rehabilitation at Dezhou Maternal and Child Health Hospital between July and December 2025. ADHD diagnoses were established by senior pediatric neurologists in accordance with the Diagnostic and Statistical Manual of Mental Disorders, Fifth Edition (DSM-5) criteria. The inclusion criteria for the ADHD group were: (1) a confirmed diagnosis of ADHD based on DSM-5 criteria; (2) age between 6 and 12 years; (3) ability to cooperate with oral examination; and (4) written informed consent provided by a parent or legal guardian. The exclusion criteria were: (1) coexisting autism spectrum disorder, intellectual disability, or other neurodevelopmental conditions that could independently affect oral health; (2) chronic systemic diseases (e.g., diabetes) known to confound oral health; (3) use of medications other than methylphenidate with known adverse effects on oral health; and (4) incomplete clinical or questionnaire data.

The control group consisted of 143 age-matched healthy children who underwent routine oral health examinations in the Department of Pediatric Stomatology during the same study period. Eligibility criteria for the control group included: (1) no history of ADHD or other neurodevelopmental disorders; (2) no chronic systemic diseases; (3) no regular use of medications known to affect oral health; and (4) complete clinical and questionnaire data.

This study protocol was approved by the Ethics Committee of Dezhou Maternal and Child Health Hospital (Ethics Approval Number: YXLL-202513). Written informed consent was obtained from the parents or legal guardians of all participants before enrollment. All participants’ personal information was handled confidentially throughout the study. The participant screening and enrollment workflow is illustrated in Supplementary File S4.

### Sample size determination

The required sample size for this case-control study was estimated using the two independent-sample proportion formula, with caries prevalence as the primary outcome. Parameters were set based on published data on oral health in children with ADHD and age-stratified caries prevalence figures for children aged 6–12 years from the Fourth National Oral Health Epidemiological Survey. The following parameters were adopted: an expected caries prevalence in the ADHD group, p₁ = 80% [12 –15]; an expected caries prevalence in the healthy control group, p₂ = 50% [19]; a two-tailed significance level α = 0.05 (Z₁₋α/₂ = 1.96); a statistical power 1 − β = 0.80 (Z₁₋β = 0.84); and an attrition correction factor f = 1.20 to accommodate potential non-cooperation or incomplete records, which are common in pediatric field studies. The sample size was calculated using the following formula:

$$\:{n} = \frac{\left(\mathrm{Z}_{1}-\alpha/2 + \mathrm{Z}_{1}-\beta\right)^2 \left[p_1\left(1 - p_1\right) + p_2\left(1 - p_2\right)\right]}{\left(p_1 - p_2\right)^2}\times{f}$$

The calculation yielded a minimum sample size of 43 participants per group (86 in total). However, several considerations motivated a substantial increase beyond this minimum. First, the study design included planned subgroup analyses stratified by age (6–7, 8–9, and 10–12 years) and gender, which require sufficient numbers in each stratum to enable valid comparisons. Second, multivariable logistic regression formed the core analytical framework to examine the associations between potential explanatory variables and oral health outcomes among children with ADHD. Following the widely accepted rule of thumb that at least 10 participants per independent variable are needed to ensure model stability and interpretability, and with a total of 10 prespecified predictor variables(age, gender, methylphenidate use, disease duration, daily brushing frequency, perfunctory toothbrushing behavior, parental supervision of toothbrushing, frequency of candy consumption, monthly household income, and parental education level), a minimum of 100 participants per group was considered appropriate for the regression analyses. Third, because participants were recruited using a hospital-based convenience sampling design, a larger sample was expected to reduce sampling error and improve the generalizability of the findings.

Considering these requirements, the minimum sample was conservatively scaled upward. The final analytic sample comprised 130 children with ADHD (mean age 9.10 ± 0.16 years; 100 males [76.9%]) and 143 healthy controls (mean age 9.25 ± 0.17 years; 103 males [72.0%]), yielding a total of 273 participants. Neither age nor gender distribution differed significantly between the two groups (both *P* > 0.05). This final sample size substantially exceeded the minimum requirements for all planned analyses — group comparisons of core oral health indicators, age- and gender-stratified subgroup analyses, and multivariable logistic regression — thereby ensuring adequate statistical power and supporting the scientific validity of the study conclusions.

### Investigation methods

#### Oral health examination

Oral examinations were conducted according to the *Basic Methods for Oral Health Surveys* (5th edition) [18] published by the World Health Organization and the protocols of the Fourth National Oral Health Epidemiological Survey [19]. The examination protocol was designed to accommodate the behavior characteristics of children with ADHD. Examinations were performed using either the knee-to-knee method or a sitting position, with the examiner positioned behind the child. Examinations were performed under artificial cold-light illumination using disposable instrument kits to assess oral health status in a standardized sequence. Two trained recorders assisted each examination session. All oral examinations were conducted by trained attending physicians with at least five years of experience in pediatric dentistry at the Department of Stomatology, Dezhou Maternal and Child Health Hospital. Prior to data collection, all examiners underwent standardized calibration training, and inter-examiner reliability was assessed using Cohen’s kappa coefficient, with values exceeding 0.70, indicating substantial agreement.

1. Caries: Caries status was assessed by visual inspection and probing. Teeth were scored as follows: 0, sound; 1, decayed (D); 2, missing due to caries (M); 3, restored (F). A score ≥ 1 indicated the presence of caries [19].
2. Plaque Index (PI): Plaque accumulation was assessed by both visual inspection and probing. Each tooth was evaluated on four surfaces: buccal, lingual, mesial, and distal. Teeth were scored as follows: 0, no plaque at the gingival margin; 1, thin plaque detected only by probing; 2, moderate plaque visible to the naked eye; 3, abundant soft plaque. The individual PI was calculated as the arithmetic mean of scores across all examined tooth surfaces. A score ≥ 2 indicated poor oral hygiene [20].
3. Modified Gingival Index (MGI): Gingival status was evaluated by visual inspection. Gingiva were scored as follows: 0, healthy gingiva; 1, mild localized inflammation; 2, moderate inflammation; 3, severe inflammation with spontaneous bleeding. A score ≥ 2 indicated gingivitis [21].
4. Cariostat caries activity test: All participants underwent caries activity testing. Specimens were collected by wiping the buccal surfaces of maxillary molars and the labial surfaces of mandibular anterior teeth near the cervical region 3–5 times with specialized caries detection swabs. Swabs were placed into reagent bottles and incubated at 37 °C in a constant-temperature incubator for 48 h. Readings were interpreted as follows: 0–0.5 indicated low caries risk, 1.0–1.5 indicated moderate caries risk, and 2.0–3.0 indicated high caries risk [22].
5. Screening for dysfunctional habits: Dysfunctional habits were evaluated using parent-reported questionnaires and clinical observation, assessing 10 indicators including mouth breathing, unilateral chewing, lip biting, and finger sucking. The presence of two or more abnormal indicators was defined as a significant abnormality [23, 24].

### Questionnaire

The questionnaire was developed based on the Fourth National Oral Health Epidemiological Survey and collected information on oral health behaviors, including children’s oral hygiene practices, toothbrushing habits, dental utilization patterns, preventive interventions such as fissure sealants and topical fluoride application, parental oral health knowledge, parental education level, and family socioeconomic status [19]. For participants in the ADHD group, additional information was collected on ADHD-related disorders, illness duration, and medication use. Following the oral examinations, face-to-face interviews were conducted with parents to collect information on risk factors associated with oral health in children with ADHD [9].

### Statistical analysis

Data processing and analysis were performed using SPSS version 26.0. Normally distributed continuous variables were presented as mean ± standard deviation (*SD*), whereas non-normally distributed data were presented as median (interquartile range, *IQR*). Categorical data were presented as frequencies (percentages). For intergroup comparisons, between-group differences in measurement data were analyzed using the independent-samples *t-test* or *Mann-Whitney U test.* Comparisons across three or more groups were performed via one-way *ANOVA* or the *Kruskal–Wallis H test.* Intergroup comparisons of categorical data were performed using the chi-square (χ²) test. The Fisher exact test was used when the expected frequency was less than 5 [25]. Two multivariate logistic regression approaches were implemented: forced-entry regression for dental caries (all five pre-specified variables were simultaneously entered regardless of univariate significance), and backward likelihood ratio stepwise selection for periodontal indicators (PI ≥ 2, MGI ≥ 2). Results were expressed as adjusted odds ratios (*aOR*) with corresponding 95% confidence intervals (*95% CI*). All statistical differences were evaluated at a significance level of *P* < 0.05. All primary multivariate results rely on Wald CIs, which tend to be overly wide under sparse categorical data, hence Firth penalized regression with profile likelihood CIs was used for sensitivity validation. For Firth’s penalized logistic regression sensitivity analyses, profile likelihood (*PL*) confidence intervals (*PL CIs*) were calculated instead of Wald confidence intervals to improve interval estimation under sparse-data scenarios [26, 27]. Standard Wald-type confidence intervals rely on asymptotic normality assumptions that are unreliable when categorical predictor subgroups contain few or zero observations, as was the case in several cells of our dataset. Firth’s penalized logistic regression with profile likelihood confidence intervals was therefore adopted to provide more reliable inference under these sparse-data conditions.

## Results

### Caries prevalence

Among the 130 children with ADHD, 105 had dental caries, corresponding to an overall caries prevalence of 80.77%. DMFT+dmft scores were not normally distributed, with a median of 3 (IQR: 2–5). The prevalence of dental caries showed a decreasing trend with increasing age, from 90.63% among children aged 6–7 years to 76.92% among those aged 10–12 years, yet the difference was not statistically significant (*P >* 0.05). The caries prevalence was significantly higher in males than in females (85.00% vs. 66.67%; *P* = 0.025). DMFT+dmft scores differed significantly between genders and among different age groups (*P* < 0.05), as shown in Table 1.

**Table 1:** Caries status among children with ADHD

Group	Total	No. of caries	Caries prevalence (%)	Statistical Analysis	Total DMFT+dmft	DMFT+dmft (Q1,Q3)	Statistical Analysis
6–7 years	32	29	90.63	*χ*^*2*^ = 2.683 *P* = 0.262	132	4 (2.25, 6)	*H* = 8.071 *P* = 0.018^*^
8–9 years	46	36	78.26		123	3 (1, 4)	
10–12 years	52	40	76.92		159	3 (1.25, 4)	
Male	100	85	85.00	*χ*^*2*^ = 4.994 *P* = 0.025*	346	4 (2, 5)	*U* = 1014.000 *P* = 0.006^*^
Female	30	20	66.67		68	2 (0, 4)	
Total	130	105	80.77	-	414	3 (2, 5)	-

In this mixed-dentition population (aged 6–12 years), DMFT was used to assess permanent teeth and dmft to assess primary teeth; overall caries experience is reported as DMFT+dmft. DMFT+dmft scores were non-normally distributed (Shapiro–Wilk test, *P* < 0.05); therefore, group comparisons were performed using the Kruskal–Wallis H test and Mann-Whitney U test. Data are presented as median (Q1, Q3), where Q1 and Q3 denote the first and third quartiles

*DMFT *decayed, missing, and filled teeth (permanent dentition), *dmft *decayed, missing, and filled teeth (primary dentition)

^*^P < 0.05 was considered statistically significant

**Fig. 1:**
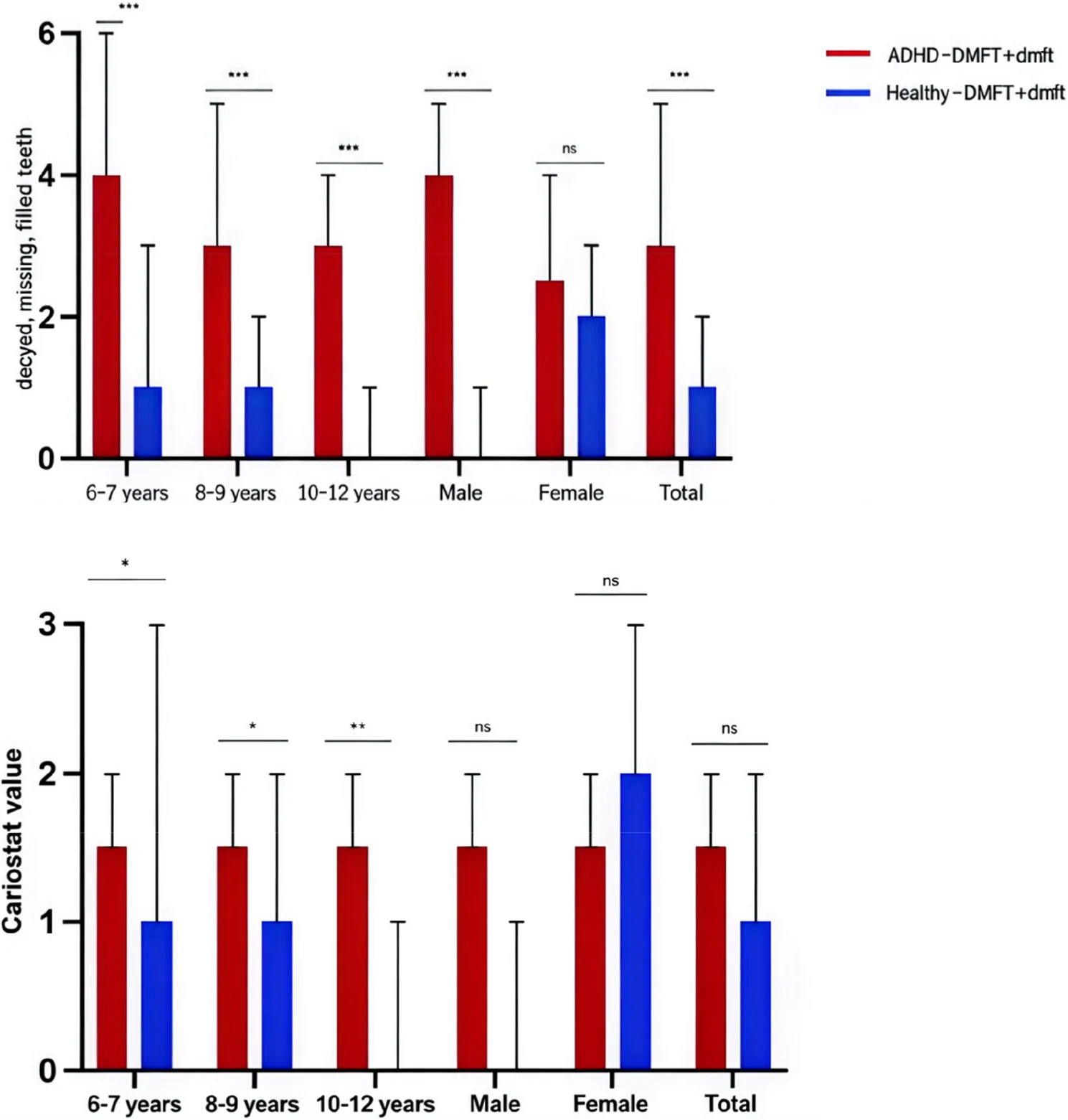
Comparison of DMFT+dmft and Cariostat values by gender and age group between the two study groups of children. Note: DMFT, decayed, missing, and filled teeth (permanent dentition); dmft, decayed, missing, and filled teeth (primary dentition); in this mixed-dentition population (aged 6–12 years), total caries experience was computed as DMFT+dmft. Cariostat, a chairside caries activity test based on the acidogenic capacity of oral microorganisms; higher values indicate greater caries risk. ADHD, attention-deficit/hyperactivity disorder. DMFT+dmft and Cariostat data were non-normally distributed (Shapiro–Wilk test, all *P* < 0.05); therefore, the Mann–Whitney U test was used for two-group comparisons (ADHD vs. Healthy controls; male vs. female) and the Kruskal–Wallis H test for comparisons across three or more groups (age strata). Data are presented as median(Q1, Q3), where Q1 and Q3 denote the first and third quartiles. Error bars represent the interquartile range (IQR).^*^P < 0.05, ^**^P < 0.01, ^***^P < 0.001; ns, not significant(*P* ≥ 0.05)

Children in the ADHD group had significantly higher overall caries prevalence (80.77% vs. 52.45%, *P* < 0.0001) and median DMFT+dmft scores than healthy controls, whereas overall Cariostat values did not differ significantly between groups (Fig. 1). Significant differences in caries prevalence were observed in the 6–7 years and 10–12 years age strata, with the largest difference in the 10–12 age group (76.92% vs. 40.00%, *P* < 0.0001); no significant difference was found in the 8–9 years age group (*P* = 0.364). Among boys, those with ADHD had significantly higher caries prevalence and DMFT+dmft scores than healthy male controls, but Cariostat values did not differ significantly. No significant differences were observed between female groups (*P* > 0.05), as detailed in Table 2; Fig. 1.

**Table 2:** Comparison of caries prevalence by gender and age in the two groups

Group		Total	No. of caries	Caries prevalence (%)	Statistical analysis
6–7 years	ADHD	32	29	90.63	*χ*^*2*^ = 7.053 *P* = 0.008^*^
	Healthy	32	20	62.50	
8–9 years	ADHD	46	36	78.26	*χ*^*2*^ = 0.824 *P* = 0.364
	Healthy	36	25	69.44	
10–12 years	ADHD	52	40	76.92	*χ*^*2*^ = 16.92 *P <* 0.0001^*^
	Healthy	75	30	40.00	
Male	ADHD	100	85	85.00	*χ*^*2*^ = 36.07 *P* < 0.0001^*^
	Healthy	103	46	44.66	
Female	ADHD	30	20	66.67	*χ*^*2*^ = 0.278 *P =* 0.598
	Healthy	40	29	72.50	
Total	ADHD	130	105	80.77	*χ*^*2*^ = 24.32 *P* < 0.0001^*^
	Healthy	143	75	52.45	

Caries prevalence was compared between the ADHD and healthy control groups stratified by gender and age group. The chi-square (χ²) test was used; Fisher’s exact test was applied when any expected cell frequency was < 5. Values are presented as n (%)

*DMFT *decayed, missing, and filled teeth (permanent dentition), *dmft *decayed, missing, and filled teeth (primary dentition), *ADHD *attention-deficit/hyperactivity disorder

^*^P < 0.05 was considered statistically significant; *P* > 0.05 indicates no significant difference

**Fig. 2:**
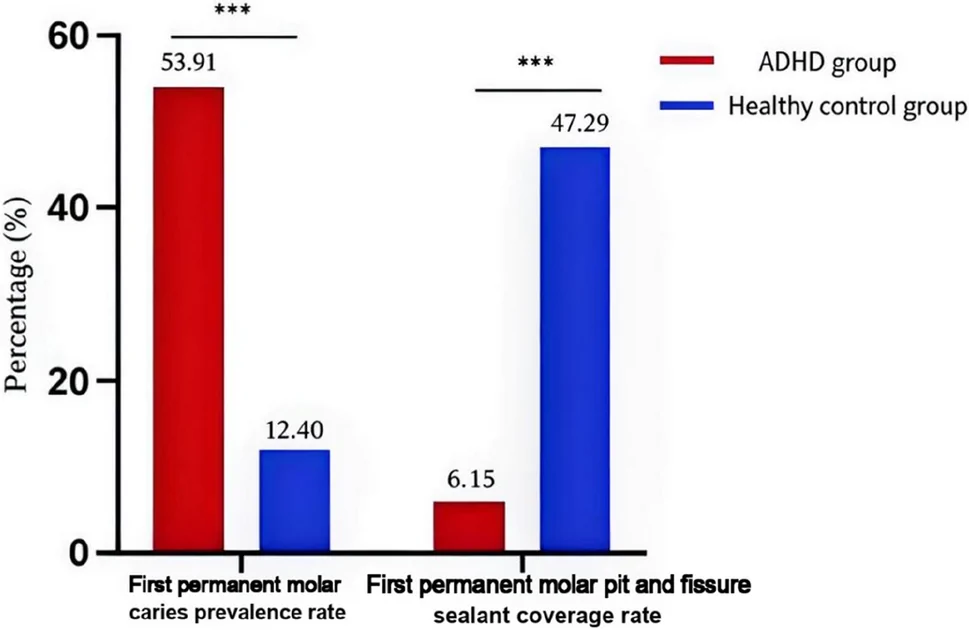
Comparison of first permanent molar caries prevalence and pit and fissure sealant coverage between children with ADHD and healthy controls. Note: Categorical rate data were analyzed with the chi-square (χ²) test. Fisher’s exact test was utilized in cases where the expected frequency of any cell was less than 5. Data are expressed as percentages (%). ADHD, attention-deficit/hyperactivity disorder. ^***^P < 0.001

Comparison of first permanent molar caries prevalence and pit and fissure sealant coverage between the two groups is presented in Fig. 2. Children with ADHD exhibited significantly higher first permanent molar caries prevalence than healthy controls (53.91% vs. 12.40%, *P* < 0.001). In contrast, pit and fissure sealant coverage was significantly lower in the ADHD group (6.15% vs. 47.29%, *P* < 0.001), suggesting marked differences in the utilization of preventive dental services between the two groups.

### Periodontal Health Status

No significant differences in the detection rates of Plaque Index (PI) ≥ 2 or Modified Gingival Index (MGI) ≥ 2 were observed among children with ADHD across age groups or genders (all *P* > 0.05). Detailed results are presented in Table 3.

**Table 3:** Comparison of Plaque Index (PI) and Modified Gingival Index (MGI) among children with ADHD by age group and gender

Group	PI < 2	PI ≥ 2	MGI < 2	MGI ≥ 2
6–7 years	5	27	11	21
	15.62%	84.38%	34.37%	65.63%
8–9 years	16	30	18	28
	34.78%	65.22%	39.13%	60.87%
10-12years	18	34	22	30
	34.61%	65.39%	42.30%	57.70%
	*H* = 5.260, *Df = 2*,* P* = 0.072		*H* = 0.489, *Df = 2*,* P* = 0.783	
Male	28	72	37	63
	28.00%	72.00%	37.00%	63.00%
Female	11	19	14	16
	36.67%	63.33%	46.67%	53.33%
	*U* = 1284.500, *Z* = -1.402, *P =* 0.161		*U* = 1270.000, *Z* = -1.449, *P =* 0.147	

PI (Plaque Index) was scored on a 4-point scale (0–3); scores ≥ 2 indicate poor oral hygiene. MGI (Modified Gingival Index) was scored on a 4-point scale (0–3); scores ≥ 2 indicate gingivitis. Both PI and MGI data were non-normally distributed; therefore, the Kruskal–Wallis H test was used for comparisons across three age groups, and the Mann–Whitney U test was used for comparisons between genders. Values are presented as n (%)

*H *Kruskal–Wallis test statistic *Df *degrees of freedom, *U *Mann–Whitney U test statistic, Z standard normal deviate, *ADHD *attention-deficit/hyperactivity disorder

^*^P < 0.05 was considered statistically significant; *P* > 0.05 indicates no significant difference

Both the Plaque Index (PI) and Modified Gingival Index (MGI) were significantly higher in the ADHD group than in the healthy controls across all age groups and genders. The ADHD group exhibited significantly higher PI scores across all strata (all *P* < 0.001) and significantly higher MGI scores across all strata (all *P* < 0.0001), as detailed in Tables 4 and 5.

**Table 4:** Comparison of Plaque Index (PI) between children with ADHD and healthy children

Group		0	1	2	3	PI M (Q1, Q3)	
6–7 years	ADHD	0	5	23	4	2 (2, 2)	*U* = 74.000 *Z* =-6.336 *P <* 0.0001
		0	15.63%	71.88%	12.50%		
	Healthy	8	23	1	0	1 (0.25, 1)	
		25.00%	71.88%	3.12%	0		
8–9 years	ADHD	0	16	27	3	2 (1, 2)	*U* = 478.500 *Z* = -3.558 *P <* 0.0001
		0	34.78%	58.70%	6.52%		
	Healthy	6	21	6	3	1 (1, 1.75)	
		16.67%	58.33%	16.67%	8.33%		
10–12 years	ADHD	0	18	32	2	2 (1, 2)	*U* = 599.000 *Z* = -7.615 *P <* 0.0001
		0	34.62%	61.54%	3.85%		
	Healthy	14	59	2	0	1 (1, 1)	
		18.67%	78.67%	2.66%	0		
Male	ADHD	0	28	63	9	2 (1, 2)	*U* = 1352.000 *Z* = -9.987 *P <* 0.0001
		0	28.00%	63.00%	9.00%		
	Healthy	22	77	3	1	1 (1, 1)	
		21.36%	74.76%	2.91%	0.97%		
Female	ADHD	0	11	19	0	2 (1, 2)	*U* = 326.000 *Z* = -3.621 *P <* 0.0001
		0	36.67%	63.33%	0		
	Healthy	6	26	6	2	1 (1, 1)	
		15.00%	65.00%	15.00%	5.00%		
Total	ADHD	0	39	82	9	2 (1, 2)	*U* = 1310.55 *Z* = -9.612 *P* < 0.0001
		0	30.00%	63.08%	6.92%		
	Healthy	28	103	9	3	1 (1, 1)	
		19.58%	72.03%	6.29%	2.10%		

The Plaque Index (PI) was scored on a 4-point scale (0 = no plaque; 1 = thin film at the gingival margin; 2 = moderate accumulation; 3 = abundant plaque). PI data were non-normally distributed; therefore, the Mann–Whitney U test was used for between-group comparisons. Values are presented as n (%) for each PI score category and as median (Q1, Q3) for the overall PI score

*U* Mann–Whitney U test statistic, *Z *standard normal deviate, *ADHD *attention-deficit/hyperactivity disorder

*P* < 0.05 was considered statistically significant

**Table 5:** Comparison of Modified Gingival Index (MGI) between children with ADHD and healthy children

Group		0	1	2	3	MGI M (Q1, Q3)	
6–7 years	ADHD	0	11	20	1	2 (1, 2)	*U* = 124.500 *Z* = -5.547 *P* < 0.0001
		0	34.38%	62.50%	3.13%		
	Healthy	15	15	2	0	1 (0, 1)	
		46.88%	46.87%	6.25%	0		
8–9 years	ADHD	1	17	25	3	1 (1, 2)	*U* = 153.000 *Z* = -6.674 *P <* 0.0001
		2.17%	36.96%	54.35%	6.52%		
	Healthy	21	15	0	0	0 (0, 1)	
		58.33%	41.67%	0	0		
10–12 years	ADHD	1	21	28	2	2 (1, 2)	*U* = 790.500 *Z* = -6.762 *P <* 0.0001
		1.92%	40.38%	53.85%	3.85%		
	Healthy	11	62	2	0	1 (1, 1)	
		14.67%	82.67%	2.67%	0		
Male	ADHD	0	37	57	6	2(1, 2)	*U* = 1538.500 *Z* = -9.489 *P <* 0.0001
		0	37.00%	57.00%	6.00%		
	Healthy	30	69	4	0	1 (0, 1)	
		29.13%	66.99%	3.88%	0		
Female	ADHD	2	12	16	0	2 (1, 2)	*U* = 201.000 *Z* = -5.157 *P <* 0.0001
		6.67%	40.00%	53.33%	0		
	Healthy	17	23	0	0	1 (0, 1)	
		42.50%	57.50%	0	0		
Total	ADHD	2	49	73	6	2 (1, 2)	*U* = 1829.00 *Z* = -8.366 *P <* 0.0001
		1.54%	37.69%	56.15%	4.62%		
	Healthy	47	92	4	0	1 (0, 1)	
		32.87%	64.34%	2.80%	0		

The Modified Gingival Index (MGI) was scored on a 4-point scale (0 = normal gingiva; 1 = mild inflammation; 2 = moderate inflammation; 3 = severe inflammation). MGI data were non-normally distributed; therefore, the Mann–Whitney U test was used for between-group comparisons. Values are presented as n (%) for each MGI score category and as median (Q1, Q3) for the overall MGI score

*U *Mann–Whitney U test statistic, *Z *standard normal deviate, *ADHD *attention-deficit/hyperactivity disorder

*P* < 0.05 was considered statistically significant

### Questionnaire survey

The ADHD group comprised 130 children with a median age of 9 years (IQR: 7.75–11). The healthy control group comprised 143 children with a median age of 10 years (IQR: 8–11). No significant differences in age or gender distribution were observed between groups (*P* > 0.05). The proportion of families with a monthly household income below CNY 6,000 was significantly higher in the ADHD group (58.46%) than in the healthy control group (16.79%) (*P* < 0.001) (Table 6 ).

**Table 6:** Demographic characteristics of children with ADHD and healthy children

	Variable	ADHD (*n* = 130)	Healthy (*n* = 143)	Statistical Test	*P*-value
Demographic characteristics	Age (years), median (IQR)	9 (7.75, 11)	10 (8, 11)	*U =* 8819.000 *Z =* -0.738	*P* = 0.460
	Gender, n (%)			*χ*^*2*^ = 0.858	*P =* 0.355
	Male	100 (76.92%)	103 (72.03%)		
	Female	30 (23.08%)	40 (27.97%)		
	Primary Caregiver, n (%)			*χ*^*2*^ = 2.202	*P* = 0.138
	Parents	117 (90.00%)	120 (83.92%)		
	Grandparents	13 (10.00%)	23 (16.08%)		
	Monthly Household Income (yuan), n (%)			*χ*^*2*^ = 50.97	*P* < 0.0001^*^
	< 6000	76 (58.46%)	24 (16.79%)		
	≥ 6000	54 (41.54%)	119 (83.22%)		
	Parental Education Level, n (%)			*χ*^*2*^ = 0.302	*P* = 0.583
	Middle/High School or Below	57 (43.85%)	58 (40.56%)		
	College/University or Above	73 (56.15%)	85 (59.44%)		

Age was non-normally distributed; therefore, the Mann–Whitney U test was used for between-group comparison and data are presented as median (IQR). For categorical variables, the chi-square (χ²) test was used; Fisher’s exact test was applied when any expected cell frequency was < 5. Categorical data are presented as n (%)

IQR interquartile range, U Mann–Whitney U test statistic,  Z standard normal deviate, *ADHD* attention-deficit/hyperactivity disorder

^*^P < 0.05 was considered statistically significant; *P* > 0.05 indicates no significant difference

Children in the ADHD group initiated toothbrushing significantly later than healthy controls; 85.38% of children in the ADHD group started brushing at 3–4 years of age, whereas 72.03% of healthy controls started before 3 years of age (*P* < 0.0001). In addition, the ADHD group had significantly shorter toothbrushing duration, with 55.38% brushing for less than 2 min compared with 23.78% in the healthy controls (*P* < 0.0001). Regarding oral health knowledge awareness, a significantly higher proportion of guardians of children in the ADHD group reported a “complete lack of understanding” of oral health knowledge (6.92% vs. 0%, *P* < 0.0001). Regarding healthcare-seeking behavior, a significantly lower proportion of children in the ADHD group had visited a dental clinic within the past 6 months (20.77% vs. 87.41%, *P* < 0.0001), and a significantly higher proportion had not sought dental care for more than 1 year (28.46% vs. 4.90%, *P* < 0.0001) (Table 7).

**Table 7:** Oral health-related behaviors in children with ADHD and healthy children

	Category	ADHD (*n* = 130)	Healthy (*n* = 143)	Statistical Test	*P*-value
Oral Hygiene Behavior	Age of Toothbrushing Initiation			/	*P* < 0.0001
	Before 3 years	3 (2.31%)	103 (72.03%)		
	3–4 years	111 (85.38%)	30 (20.98%)		
	4–5 years	14 (10.77%)	9 (6.29%)		
	5–6 years	2 (1.54%)	1 (0.70%)		
	Not started	0 (0)	0(0)		
	Daily Frequency of Toothbrushing			*χ*^*2*^ = 0.8305	*P =* 0.3621
	≥ 2 times	25 (19.23%)	34 (23.78%)		
	< 2 times	105 (80.77%)	109 (76.22%)		
	Toothbrushing Duration			*χ*^*2*^ = 28.64	*P* < 0.0001
	< 2 min	72 (55.38%)	34 (23.78%)		
	≥ 2 min	58 (44.62%)	109 (76.22%)		
Oral Health Knowledge	Fluoride toothpaste prevents caries	110 (84.62%)	80 (55.94%)	*χ*^*2*^ = 88.39	*P* < 0.0001
	Correct use of floss/interdental brushes	39 (30.00%)	39 (27.27%)		
	Importance of regular dental check-ups	35 (26.92%)	126 (88.11%)		
	Relationship between high-sugar diet and caries	35 (26.92%)	128 (89.51%)		
	Complete lack of understanding	9 (6.92%)	0 (0)		
Healthcare-Seeking Behavior	Time of Last Dental Visit			*χ*^*2*^ = 126.9	*P* < 0.0001
	Within 6 months	27 (20.77%)	125 (87.41%)		
	6–12 months	18 (13.85%)	8 (5.59%)		
	> 12 months	37 (28.46%)	7 (4.90%)		
	Never visited	48 (36.92%)	3 (2.10%)		

All variables were categorical and compared between groups using the chi-square (χ²) test. Fisher’s exact test was applied when any expected cell frequency was < 5. Values are presented as n (%)

*ADHD *attention-deficit/hyperactivity disorder

^*^P < 0.05 was considered statistically significant; *P* > 0.05 indicates no significant difference

The proportion of children with high caries risk was significantly higher in the ADHD group (42.31%) than in the healthy control group (13.99%), whereas the proportion with low caries risk was significantly lower in the ADHD group (1.54%) than in the healthy control group (13.99%). A significant difference in Cariostat scores was observed between the two groups: the ADHD group had a median of 1.5 (IQR: 1.5–2.0), compared with 1.5 (IQR: 1.5–1.5) in the healthy controls (*P* < 0.0001) (Table 8).

**Table 8:** Cariostat test results for ADHD children and healthy children

	Category	ADHD (*n* = 130)	Healthy (*n* = 143)	Statistical Test	*P*
Caries Risk Assessment	Low risk, n (%)	2 (1.54%)	20 (13.99%)	*χ*^*2*^ = 35.64	*P* < 0.0001
	Moderate risk, n (%)	73 (56.15%)	103 (72.03%)		
	High risk, n (%)	55 (42.31%)	20 (13.99%)		
	Cariostat score, median (IQR)	1.5 (1.5, 2)	1.5 (1.5, 1.5)	*U* = 5942.500 *Z =* -6.051	*P* < 0.0001

The Cariostat test is a chairside caries activity test based on the acidogenic capacity of oral microorganisms; higher scores indicate greater caries risk. Cariostat values were non-normally distributed; therefore, the Mann–Whitney U test was used for between-group comparison of overall scores and the chi-square (χ²) test for categorical comparisons of risk levels. Fisher’s exact test was applied when any expected cell frequency was < 5. Values are presented as n (%) for categorical variables and as median (Q1, Q3) for Cariostat scores

*U *Mann–Whitney U test statistic, *ADHD *attention-deficit/hyperactivity disorder

^*^P < 0.05 was considered statistically significant

### Influencing factors of oral health conditions in children with ADHD

Univariate analysis revealed that gender (*P* = 0.025), primary caregiver (*P* = 0.013), candy consumption frequency (*P* < 0.0001), parental education level (*P* = 0.008), and methylphenidate medication use (*P* < 0.0001) were significantly associated with dental caries in children with ADHD (Tables 9 and 10).

**Table 9:** Univariate analysis of factors related to dental caries (DMFT+dmft > 0) in children with ADHD

Factor	Total	Number of Children with Dental Caries (DMFT+dmft > 0), *n* (%)	χ^2^	*P*
Gender			4.994	0.025^*^
Male	100	85 (85.00%)		
Female	30	20 (66.67%)		
Age Group			2.683	0.262
6–7 years	32	29 (90.63%)		
8–9 years	46	36 (78.26%)		
10–12 years	52	40 (76.92%)		
Primary Caregiver			6.175	0.013^*^
Parents	117	97 (82.91%)		
Grandparents	13	7 (53.85%)		
Daily Toothbrushing Frequency			0.012	0.914
≥ 2 times	25	20 (80.00%)		
< 2 times	105	85 (80.95%)		
Toothbrushing Duration			2.965	0.085
≥ 2 min	58	43 (74.14%)		
< 2 min	72	62 (86.11%)		
Toothbrushing Supervision			0.599	0.439
Regular	45	38 (84.44%)		
Irregular	85	67 (78.82%)		

Univariate associations between each factor and the presence of dental caries (DMFT+dmft > 0) were examined using the chi-square (χ²) test. Fisher’s exact test was applied when any expected cell frequency was < 5

*DMFT *decayed, missing, and filled teeth (permanent dentition), *dmft *decayed, missing, and filled teeth (primary dentition), *ADHD *attention-deficit/hyperactivity disorder

^*^P < 0.05 was considered statistically significant

**Table 10:** Univariate analysis of factors related to dental caries (DMFT+dmft > 0) in children with ADHD

Factor	Total	Number of Children with Dental Caries (DMFT+dmft > 0)	χ^2^	*P*
Candy Consumption Frequency			69.64	< 0.0001^*^
Frequent (often)	104	99 (95.19%)		
Infrequent (rarely)	26	6 (23.08%)		
Monthly Household Income			1.395	0.238
< 6000 yuan	76	64 (84.21%)		
≥ 6000 yuan	54	41 (75.93%)		
Parental Education Level, n (%)			7.149	0.008^*^
Middle/High school or below	57	52 (91.23%)		
College/University or above	73	53 (72.60%)		
Medication Use (ADHD Treatment)			44.10	< 0.0001^*^
Yes	101	94 (93.07%)		
No	29	11 (37.93%)		
Disease Duration Group			0.186	0.667
Short-term (≤ 3 years)	73	58 (79.45%)		
Long-term (> 3 years)	57	47 (82.46%)		
Oral Health Knowledge Awareness			2.648	0.618
Fluoride toothpaste prevents caries	110	89(80.91%)		
Correct use of floss/interdental brushes	39	33(84.62%)		
Importance of regular dental check-ups	35	28(80.00%)		
Relationship between high-sugar diet and caries	35	30(85.71%)		
Complete lack of understanding	9	9 (100%)		

See Table 9 for a complete description of the statistical methods. Univariate associations were examined using the chi-square (χ²) test or Fisher’s exact test as appropriate

*DMFT* decayed, missing, and filled teeth (permanent dentition); *dmft* decayed, missing, and filled teeth (primary dentition); *ADHD* attention-deficit/hyperactivity disorder

^*^P < 0.05 was considered statistically significant

**Fig. 3:**
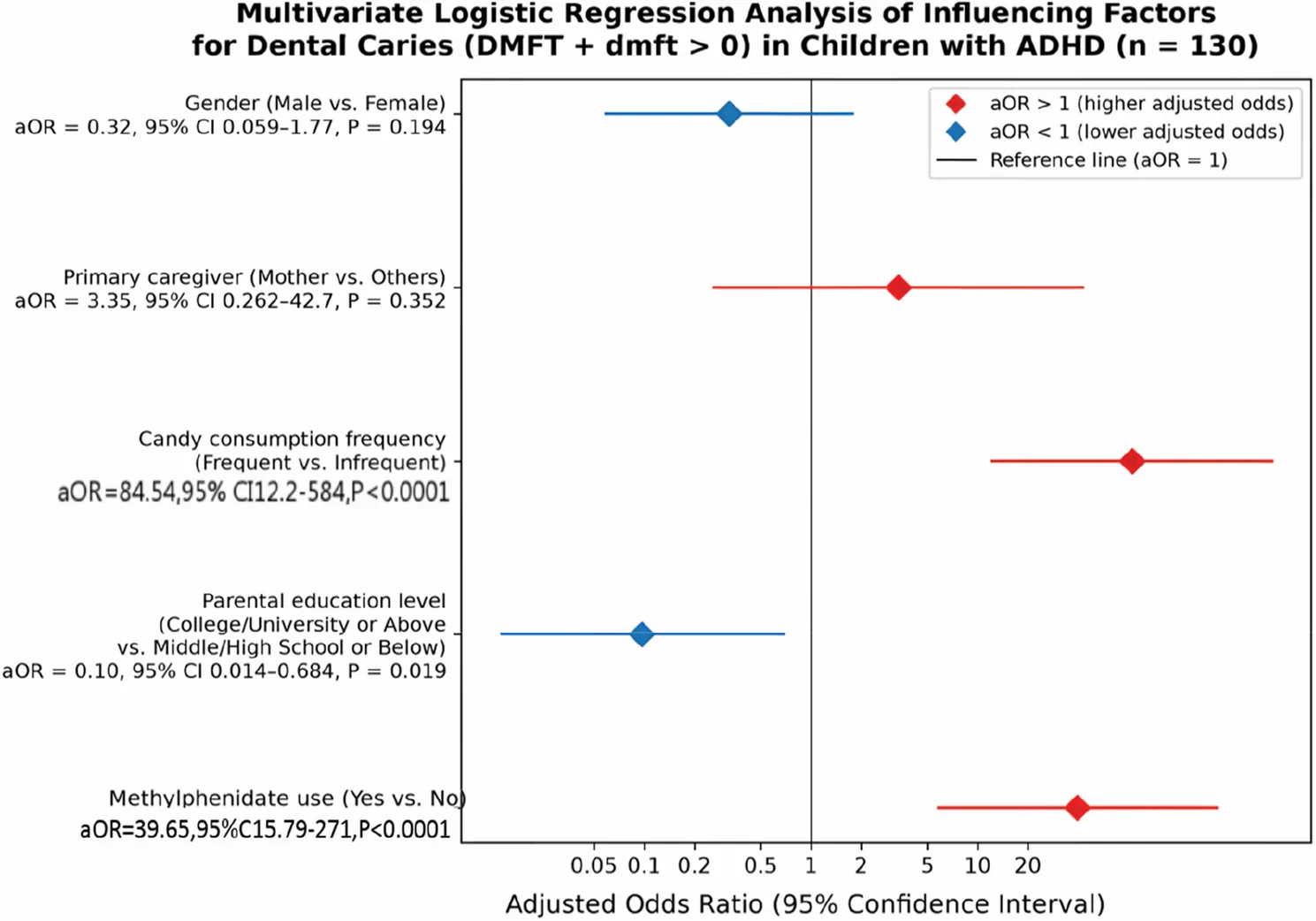
Multivariate logistic regression analysis forest plot of influencing factors for dental caries (DMFT+dmft > 0) in children with ADHD. Note: Model specification: Dependent variable, presence of dental caries (DMFT + dmft > 0); reference: DMFT + dmft = 0. All pre-specified variables (gender, primary caregiver, candy consumption frequency, parental education level, and methylphenidate use) were entered into the multivariate model using the enter method. Frequent candy consumption (reference: infrequent candy consumption; *aOR* = 84.54, *95% CI*: 12.2–584, *P* < 0.001) and methylphenidate use (reference: no medication; aOR = 39.65, 95% CI: 15.79–271, *P* < 0.0001) were associated with higher adjusted odds of dental caries. Parental education level (reference: middle/high school or below; aOR = 0.10, 95% CI: 0.014–0.684, *P* = 0.019) was associated with lower adjusted odds of dental caries. No significant statistical pattern was observed for gender or primary caregiver. DMFT, Decayed, Missing, Filled Teeth (permanent teeth); dmft, decayed, missing, filled primary teeth; aOR, adjusted odds ratio; CI, confidence interval; ADHD, attention-deficit/hyperactivity disorder. The vertical reference line indicates *aOR* = 1 (no association). X-axis uses logarithmic scale. A *P*-value < 0.05 was considered statistically significant. Due to sparse subgroup cell counts and quasi-complete separation, the 95% confidence intervals for several aOR estimates are extremely wide. The absolute magnitude of these aOR values should be interpreted with caution; the primary focus should be on the direction and statistical significance of the associations

After adjusting for confounders, multivariate logistic regression using the enter method was performed. Frequent candy consumption (reference: infrequent candy consumption) was associated with higher adjusted odds of dental caries (*aOR* = 84.54, *95% CI*: 12.2–584, *P* < 0.0001). Methylphenidate use (reference: no medication) was associated with higher adjusted odds of dental caries *(aOR* = 39.65, *95% CI*: 15.79–271, *P* < 0.0001). Parental education level (reference: middle/high school or below) was associated with lower adjusted odds of dental caries (*aOR* = 0.10, *95% CI*: 0.014–0.684, *P* = 0.019). No significant statistical pattern was observed for gender (*aOR* = 0.32, *95% CI*: 0.059–1.77, *P* = 0.194) or primary caregiver (*aOR* = 3.35, *95% CI*: 0.262–42.7, *P* = 0.352). Detailed results are illustrated in Fig. 3. The regression model for dental caries exhibited quasi-complete separation, stemming from sparse and highly imbalanced cell counts across categorical subgroups. Firth’s penalized logistic regression was implemented as a sensitivity analysis. Under Firth penalization, frequent candy consumption was associated with the highest odds of dental caries (*penalized OR* = 37.27, *95% PL CI*: 8.50–237.75, *P* < 0.0001), followed by methylphenidate use (*penalized OR* = 22.13, *95% PL CI*: 5.12–132.10, *P* < 0.0001), while higher parental education was associated with lower odds (*penalized OR* = 0.153, *95% PL CI*: 0.022–0.738, *P* < 0.0001). Gender (*penalized OR* = 0.360, *95% PL CI*: 0.069–1.675, *P* = 0.194) and primary caregiver (*penalized OR* = 1.859, *95% PL CI*: 0.084–27.96, *P* = 0.352) showed no significant association. The penalized estimates were attenuated toward the null compared with standard logistic regression, consistent with the expected behavior of Firth’s method under sparse data conditions. The extremely wide confidence intervals reflect poor precision in estimating the exact magnitude of these associations (see Supplementary Table S3).

Regarding poor oral hygiene (PI ≥ 2), significant associated factors (*P* < 0.05) included perfunctory toothbrushing (*P =* 0.0003), frequent forgetting to brush (*P* = 0.0005), daily toothbrushing frequency (*P* = 0.0003), supervision during toothbrushing (*P* = 0.009), parental education (*P =* 0.006), monthly household income (*P =* 0.0001), and oral health knowledge awareness (*P* = 0.017). Regarding gingivitis (MGI ≥ 2), significant associated factors (*P* < 0.05) included frequent forgetting to brush (*P* = 0.041), daily toothbrushing frequency (*P* = 0.0002), supervision during toothbrushing (*P* < 0.0001), monthly household income (*P* = 0.0003), and disease duration (*P* < 0.0001) (Tables 11 and 12).

**Table 11:** Univariate analysis of periodontal-related influencing factors in children with ADHD

Factor	Total	Poor Oral Hygiene (PI ≥ 2), *n* (%)	χ^2^	*P*	Gingivitis (MGI ≥ 2), *n* (%)	χ^2^	*P*
Gender			0.825	0.363		0.905	0.342
Male	100	72 (72.00%)			63 (63.00%)		
Female	30	19 (63.33%)			16 (53.33%)		
Age Group			4.177	0.124		0.523	0.770
6–7 years	32	27 (84.38%)			21 (65.63%)		
8–9 years	46	30 (65.22%)			28 (60.87%)		
10–12 years	52	34 (65.38%)			30 (57.69%)		
Toothbrushing Perfunctoriness			13.21	0.0003		0.275	0.600
Often	100	78 (78.00%)			62 (62.00%)		
Rarely	30	13 (43.33%)			17 (56.67%)		
Forgetting to Brush Teeth			11.96	0.0005^*^		4.160	0.041^*^
Often	99	77 (77.78%)			65 (65.66%)		
Rarely	31	14 (45.16%)			14 (45.16%)		
Daily Toothbrushing Frequency			13.27	0.0003^*^		13.94	0.0002^*^
≥ 2 times	25	10 (40.00%)			7 (28.00%)		
< 2 times	105	81 (77.14%)			72 (68.57%)		

Oral health knowledge was evaluated with multiple-choice items. Univariate comparisons were conducted using the Pearson chi-square test; Fisher’s exact test would be applied if the expected cell frequency was less than 5

*PI *Plaque Index, *MGI *Modified Gingival Index, *ADHD *attention-deficit/hyperactivity disorder

All variables with univariate *P* < 0.05 were included in independent multivariate binary logistic regression models via the backward likelihood ratio method for poor oral hygiene (PI ≥ 2) and gingivitis (MGI ≥ 2), respectively. ^*^P < 0.05 was defined as statistical significance

**Table 12:** Univariate analysis of periodontal-related influencing factors in children with ADHD

Factor	Total	Poor Oral Hygiene (PI ≥ 2), *n* (%)	χ^2^	*P*	Gingivitis (MGI ≥ 2), *n* (%)	χ^2^	*P*
Toothbrushing Duration			1.921	0.166		0.203	0.653
< 2 min	72	54 (75.00%)			45(62.50%)		
≥ 2 min	58	37 (63.79%)			34(58.62%)		
Toothbrushing Supervision			6.838	0.009^*^		25.39	< 0.0001^*^
Regular	45	25 (55.56%)			14(31.11%)		
Irregular	85	66 (77.65%)			65(76.47%)		
Candy Frequency			0.330	0.566		0.976	0.323
Often	104	74(71.15%)			61(58.65%)		
Rarely	26	17 (65.38%)			18(69.23%)		
Monthly Household Income			14.49	0.0001^*^		12.80	0.0003^*^
< 6000 yuan	76	63(82.89%)			56(73.68%)		
≥ 6000 yuan	54	28(51.85%)			23(42.59%)		
Parental Education			7.500	0.006^*^		1.481	0.224
Middle School or Below	57	47 (82.46%)			38(66.67%)		
College or Above	73	44 (60.27%)			41(56.16%)		
Medication Use (Methylphenidate)			2.302	0.129		0.026	0.871
Yes	101	74(73.27%)			61(60.40%)		
No	29	17(58.62%)			18(62.07%)		
Disease Duration Group			1.430	0.232		23.40	< 0.0001^*^
Short-term (≤ 3 years)	73	48(65.75%)			31(42.47%)		
Long-term (> 3 years)	57	43(75.44%)			48(84.21%)		
Oral Knowledge			12.08	0.017^*^		3.761	0.439
Fluoride toothpaste prevents caries	110	73 (66.36%)			62(56.36%)		
Correct use of floss/interdental	39	20 (51.28%)			22(56.41%)		
Importance of regular dental check-ups	35	17 (48.57%)			16(45.71%)		
High-sugar diet and caries relationship	35	18 (51.43%)			17(48.57%)		
Complete lack of understanding	9	9 (100%)			7(77.78%)		

Oral health knowledge was evaluated with multiple-choice items. Univariate comparisons were conducted using the Pearson chi-square test; Fisher’s exact test would be applied if the expected cell frequency was less than 5

*PI* Plaque Index, *MGI *Modified Gingival Index, *ADHD *attention-deficit/hyperactivity disorder

All variables with univariate *P* < 0.05 were included in independent multivariate binary logistic regression models via the backward likelihood ratio method for poor oral hygiene (PI ≥ 2) and gingivitis (MGI ≥ 2), respectively. ^*^P < 0.05 was defined as statistical significance

**Fig. 4:**
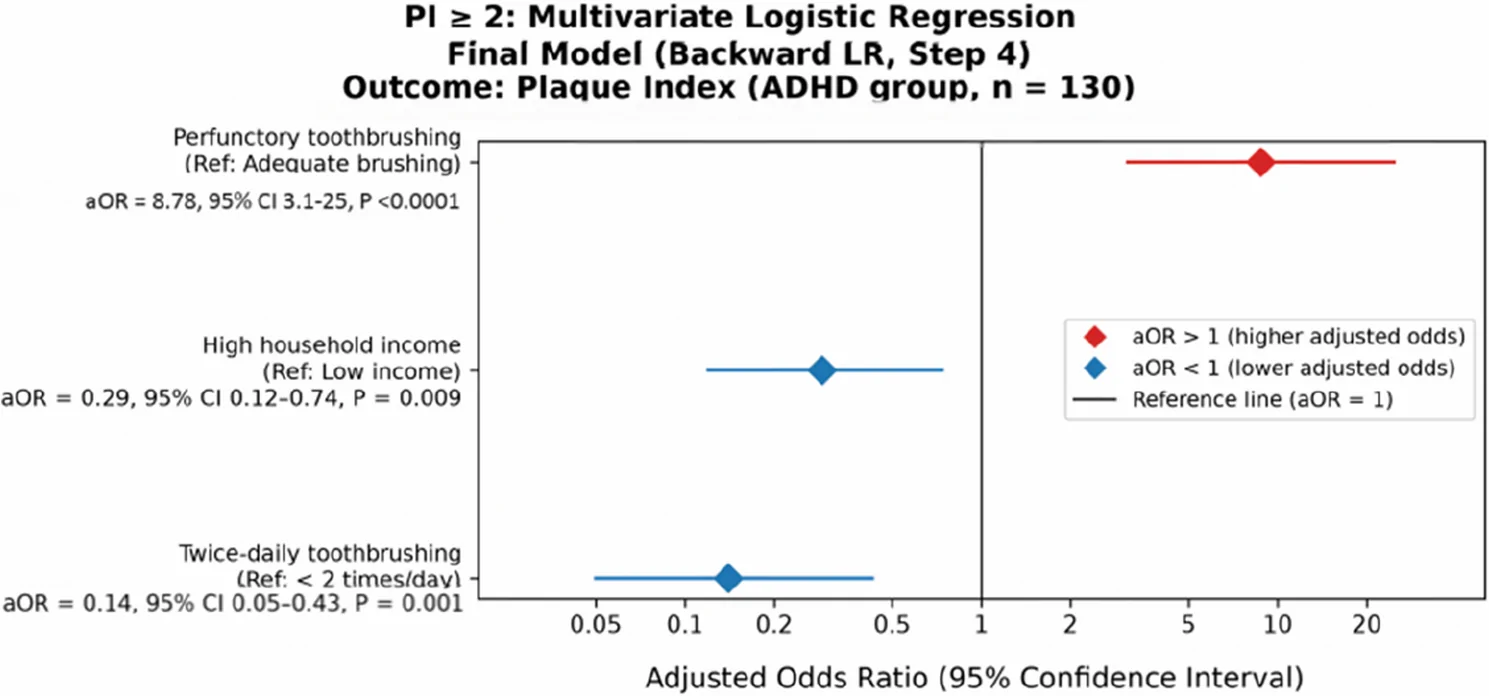
Multivariate logistic regression analysis forest plot of influencing factors for poor oral hygiene (PI ≥ 2) in children with ADHD. Note: Dependent variable, poor oral hygiene (PI ≥ 2; reference: PI < 2). Variables with *P* < 0.05 in univariate analysis (perfunctory toothbrushing, frequent forgetting to brush teeth, daily toothbrushing frequency, supervision during toothbrushing, oral health knowledge awareness, and monthly household income) were entered into the initial multivariate model. Backward likelihood ratio (LR) selection was applied to build the final model. Perfunctory toothbrushing (reference: thorough brushing; *aOR* = 8.78, *95% CI*: 3.12–24.71, *P* < 0.0001) was associated with higher adjusted odds of poor oral hygiene. Monthly household income (reference: lower income; *aOR* = 0.29, *95% CI*: 0.12–0.74, *P* = 0.009) and toothbrushing ≥ 2 times per day (reference: toothbrushing < 2 times per day; *aOR* = 0.14, *95% CI*: 0.05–0.43, *P* = 0.001) were associated with lower adjusted odds of poor oral hygiene. PI, Plaque Index (scored 0–3; ≥ 2 indicates poor oral hygiene); aOR, adjusted odds ratio; CI, confidence interval; ADHD, attention-deficit/hyperactivity disorder; LR, likelihood ratio. The vertical reference line indicates *aOR* = 1 (no association). X-axis uses logarithmic scale. A *P*-value < 0.05 was considered statistically significant

After adjusting for confounders, multivariate logistic regression (backward LR) was performed. Perfunctory toothbrushing was retained in the final model, associated with higher adjusted odds of poor oral hygiene (PI ≥ 2), with children who reported perfunctory brushing having 8.78-fold higher adjusted odds compared with children with thorough brushing habits (*aOR* = 8.78, *95% CI*: 3.12–24.71, *P* < 0.0001). Conversely, higher household income (*aOR* = 0.29, *95% CI*: 0.12–0.74, *P* = 0.009) and twice-daily toothbrushing (*aOR* = 0.14, *95% CI*: 0.05–0.43, *P* = 0.001) were retained in the final model, associated with lower adjusted odds of poor oral hygiene (PI ≥ 2). Detailed results are illustrated in Fig. 4. Firth’s penalized logistic regression was adopted for sensitivity testing to address potential estimation bias from quasi-complete separation under sparse data conditions. Standard and Firth estimates were highly consistent for PI. Under Firth penalization, perfunctory toothbrushing was associated with the highest odds of PI ≥ 2 (penalized *OR* = 8.08, *95% PL CI*: 3.05–23.08, *P* < 0.001), while high income (*penalized OR* = 0.31, *95% PL CI*: 0.12–0.75, *P* = 0.009) and brushing ≥ 2/day (*penalized OR* = 0.15, *95% PL CI*: 0.05–0.44, *P* < 0.001) were associated with lower odds. No evidence of separation issues was detected in this model (see Supplementary Table S3).

**Fig. 5:**
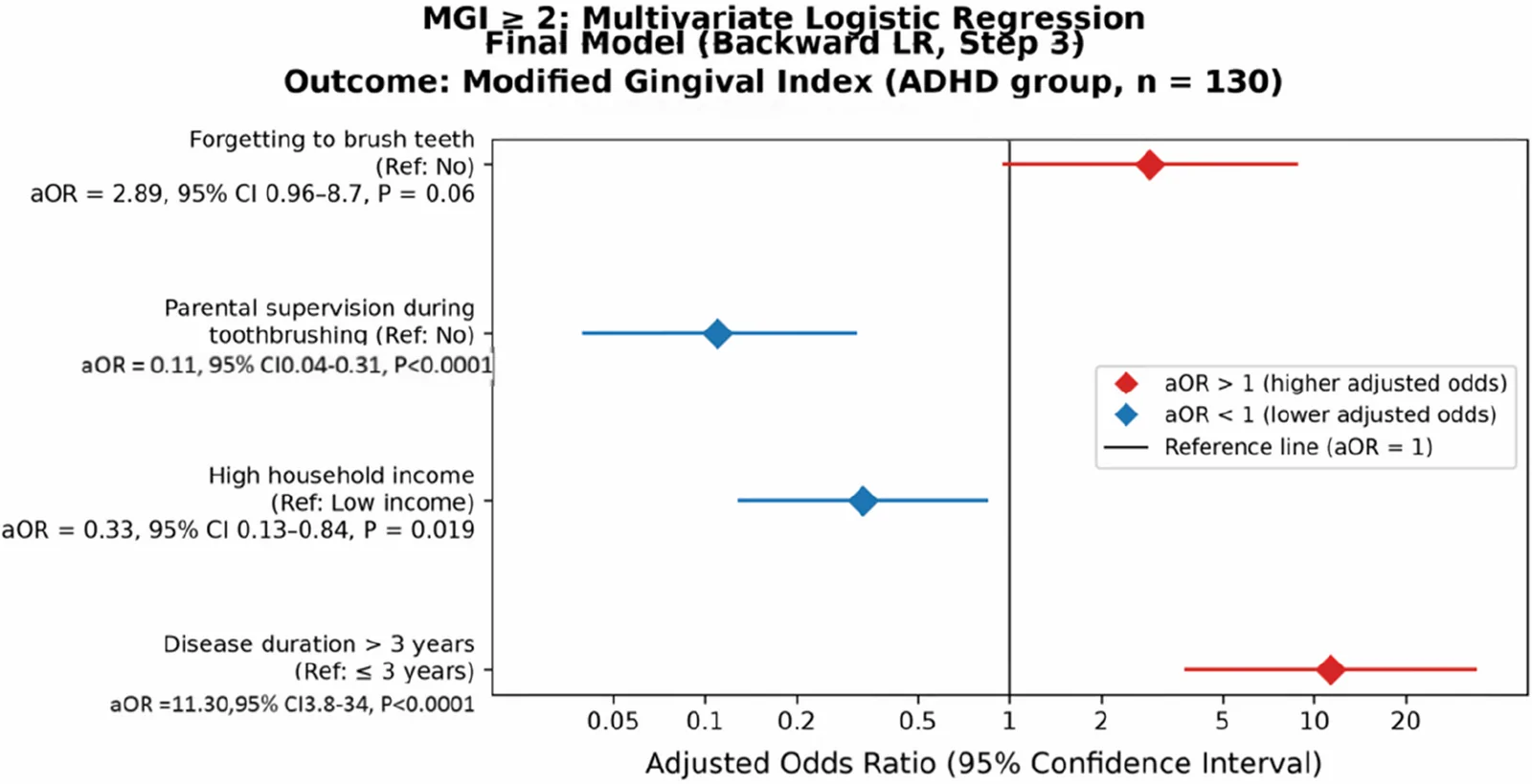
Forest plot of multivariate logistic regression analysis for influencing factors associated with gingivitis (MGI ≥ 2) in children with ADHD. Note: Dependent variable, gingivitis (MGI ≥ 2; reference: MGI < 2). Variables with *P* < 0.05 in univariate analysis (perfunctory toothbrushing, frequent forgetting to brush teeth, daily toothbrushing frequency, supervision during toothbrushing, monthly household income, and disease duration) were entered into the initial multivariate model. Backward likelihood ratio (LR) selection was applied to build the final model. Disease duration > 3 years (reference: ≤ 3 years; *aOR* = 11.30, *95% CI*: 3.78–33.75, *P* < 0.0001) was associated with higher adjusted odds of gingivitis. Toothbrushing supervision (reference: no or inadequate supervision; *aOR* = 0.11, *95% CI*: 0.04–0.31, *P* < 0.0001) and monthly household income (reference: lower income; *aOR* = 0.33, *95% CI*: 0.13–0.84, *P* = 0.019) were associated with lower adjusted odds of gingivitis. MGI, Modified Gingival Index (scored 0–3; ≥ 2 indicates gingivitis); aOR, adjusted odds ratio; CI, confidence interval; ADHD, attention-deficit/hyperactivity disorder; LR, likelihood ratio. The vertical reference line indicates *aOR* = 1 (no association). X-axis uses logarithmic scale. A P-value < 0.05 was considered statistically significant. Due to sparse subgroup cell counts and quasi-complete separation, the 95% confidence intervals for several aOR estimates are extremely wide. The absolute magnitude of these aOR values should be interpreted with caution; the primary focus should be on the direction and statistical significance of the associations

After adjusting for confounders, multivariate logistic regression (backward LR) was performed. Disease duration > 3 years was retained in the final model, associated with higher adjusted odds of gingivitis (MGI ≥ 2), with children whose disease duration exceeded 3 years having 11.30-fold higher adjusted odds than those with shorter disease duration (*aOR* = 11.30, *95% CI*: 3.78–33.75, *P* < 0.0001). Forgetting to brush teeth was retained in the final model, although the result did not reach the conventional significance threshold (*aOR* = 2.89, *95% CI*: 0.96–8.73, *P* = 0.060). Parental supervision of toothbrushing (*aOR* = 0.11, *95% CI*: 0.04–0.31, *P* < 0.0001) and higher household income (*aOR* = 0.33, *95% CI*: 0.13–0.84, *P* = 0.019) were retained in the final model, associated with lower adjusted odds of gingivitis (MGI ≥ 2). Detailed results are presented in Fig. 5. Firth’s penalized logistic regression was adopted for sensitivity testing to address potential estimation bias from quasi-complete separation under sparse data conditions. The penalized model yielded the following estimates: disease duration > 3 years (*penalized OR* = 9.70, *95% PL CI*: 3.65–30.12), frequent forgetting to brush teeth (*penalized OR* = 2.711, *95% PL CI*: 0.946–8.174), regular parental toothbrushing supervision (*penalized OR* = 0.126, *95% PL CI*: 0.045–0.319), and higher household income (*penalized OR* = 0.349, *95% PL CI*: 0.138–0.850). The direction of associations and statistical significance patterns were comparable to those obtained from the primary analysis. However, the penalized estimates are attenuated toward the null, and the exact effect sizes remain uncertain given the wide confidence intervals arising from sparse subgroup data (see Supplementary Table S3).

## Discussion

This study systematically investigated oral health status of children with ADHD and identified factors associated with adverse oral health in this understudied population. Oral health constitutes a critical component of overall health during childhood [1], and the present findings indicate that children with ADHD bear a substantially higher burden of dental caries and poorer gingival health than age- and gender-matched healthy peers [12–15]. The following sections discuss the observed associations in the context of existing evidence, with emphasis on potential mechanisms and clinical implications. Dental caries is the most prevalent chronic oral disorder in children, with a multifactorial pathogenesis involving oral microbial dysbiosis, dietary patterns, and oral hygiene behaviors. Beyond pain and local infection, severe caries has also been linked to systemic comorbidities [2]. In children, gingival disease mainly presents as gingivitis, with gingival bleeding as a typical clinical sign [4]. Accordingly, the oral health profile of children with ADHD exhibits distinctive features and warrants increased clinical and public health attention.

Although current research predominantly focuses on pharmacological and behavioral therapies for ADHD, comparatively little attention has been paid to oral health-related consequences of ADHD [12–17]. Recent international literature includes only a limited number of studies investigating how ADHD symptoms affect children’s oral health [16, 17]. These studies are fragmented and lack methodological rigor [12], and most are derived from specific populations and cultural contexts, limiting their generalizability. To date, there is a lack of systematic epidemiological survey data on the oral health status of children with ADHD in China, and the underlying association patterns and key influencing factors remain unclear. Given the potential differences in educational approaches, oral healthcare services, dietary habits, and ADHD diagnosis and management across countries, population-based evidence from China is needed to inform context-specific preventive and clinical strategies.

Core ADHD symptoms, including inattention, hyperactivity, and impulsivity, substantially compromise children’s capacity to maintain effective oral hygiene practices [13]. In this study, children with ADHD manifested poor hygiene behaviors, including insufficient brushing duration, low daily brushing frequency, and inadequate parental supervision, all of which have been consistently linked to caries risk in previous studies [11, 28, 29]. The observed gender disparity—with male children with ADHD exhibiting higher DMFT+dmft scores than their female counterparts—may reflect the greater severity of attention deficits and impulsive behaviors reported among boys with ADHD [10], which may adversely affect both oral hygiene practices and dietary self-regulation. Consistent with this explanation, frequent candy consumption was associated with higher adjusted odds of dental caries among children with ADHD. This supports the hypothesis that ADHD-related impulsivity may be associated with cariogenic dietary behaviors. In addition to individual-level factors, reduced access to preventive dental care may have contributed to the poorer oral health observed in children with ADHD. Children with ADHD in this cohort were considerably less likely to receive regular dental check-ups compared with healthy controls and consequently had lower rates of restorative treatment and preventive fissure sealant placement, resulting in missed opportunities for early detection and intervention of dental disease.

Periodontal health is fundamentally dependent on effective plaque control, which in turn requires regular and properly performed toothbrushing [20, 30]. However, the core symptoms of ADHD, particularly inattention, hyperactivity, and impulsivity, may hinder the consistent performance of daily oral hygiene behaviors [10]. In this study, perfunctory toothbrushing was retained in the final model, associated with higher adjusted odds of poor oral hygiene (PI ≥ 2) among children with ADHD. This finding lends support to the hypothesis that ADHD-related behavioral patterns may be associated with suboptimal plaque control. Notably, the majority of children with ADHD in this cohort were primarily cared for by their parents rather than by grandparents. Consistent with this observation, insufficient parental supervision of toothbrushing was retained in the final model, associated with higher adjusted odds of gingivitis (MGI ≥ 2) among children with ADHD. This finding suggests that caregiver involvement in oral hygiene practices may be linked to gingival status in children with ADHD.

Methylphenidate is a first-line central nervous system stimulant used in the clinical treatment of ADHD in children and effectively improves core symptoms such as inattention, hyperactivity, and impulsivity [33]. However, while exerting its therapeutic effects, this drug may also induce systemic and local adverse reactions, among which oral-related adverse effects are relatively common [34]. In the present study, methylphenidate use was associated with higher adjusted odds of dental caries among children with ADHD. This association is biologically plausible given the established side-effect profile of stimulant medications, which can reduce salivary flow, increase bruxism frequency, and alter dietary preferences [11]. These findings suggest that children with ADHD receiving methylphenidate may benefit from intensified oral health monitoring and preventive care as part of their comprehensive management.

To address potential estimation bias arising from quasi-complete separation within sparse subgroup cell counts, Firth’s penalized logistic regression was performed as a sensitivity analysis for all three oral outcome models. This penalized likelihood method mitigates small-sample bias induced by imbalanced categorical predictors. Across all three models, the direction of associations and statistical significance patterns were comparable to those obtained from conventional logistic regression. However, the penalized estimates are attenuated toward the null, and the exact magnitude of the adjusted odds ratios should be interpreted with caution given the wide confidence intervals arising from sparse subgroup data. These sensitivity analyses support, rather than verify, the robustness of the observed associations. Nevertheless, sparse subgroup data generates broad confidence intervals, limiting reliable quantification of effect magnitudes regardless of penalization. These findings should be considered hypothesis-generating rather than confirmatory, and future studies with larger sample sizes are needed to obtain more precise effect estimates. 

Family socioeconomic status (SES) acts as a core social factor shaping pediatric physical health trajectories [31]. Consistent with previous research focused on families caring for neurodevelopmentally impaired children, children with ADHD in this study were more likely to come from households with lower income and lower parental educational attainment [32]. Multivariate results further suggest that SES remained associated with adverse oral health outcomes after adjustment for behavioral and clinical covariates. Limited financial resources may reduce regular access to preventive dental checkups, fluoride supplies, and routine professional care, thereby compounding the challenges associated with ADHD- related behavioral characteristics. The combination of economic hardship and neurobehavioral dysfunction may compound challenges in maintaining adequate daily plaque control, potentially contributing to progressive dental lesions and delayed clinical intervention.

Although both household income and parental education are widely used indicators of SES, only household income remained significant in the final regression models, suggesting that economic resources may be more closely associated with access to preventive dental care than educational attainment alone. Collectively, these observations suggest that improving oral health in children with ADHD may require both behavioral interventions and measures that reduce socioeconomic barriers to preventive dental care.

Oral examination findings revealed that children with ADHD not only had a significantly higher burden of untreated oral disease compared with healthy children, but their behavioral characteristics also posed substantial challenges for clinicians during the diagnostic and therapeutic process [35]. Compared with healthy children, children with ADHD presented with more advanced oral disease and received lower rates of standardized restorative procedures and periodontal therapy. During examinations, some children with ADHD exhibited obvious symptoms of inattention, hyperactivity, and defiant behaviors [36], which hindered the smooth implementation of conventional oral diagnostic and therapeutic procedures and may have contributed to delays in treatment. These findings suggest that interventions for children with ADHD should not be limited to “medical consultation recommendations”; rather, a comprehensive and actionable behavioral guidance and prevention system spanning clinical practice, family, and community settings may be warranted [37].

The oral health of children with ADHD remains an understudied aspect of ADHD management in the existing literature, where research efforts have predominantly focused on pharmacological and behavioral interventions [38–40]. By systematically examining oral health status and its determinants in Chinese children with ADHD, this study provides population-specific evidence that improves the understanding of ADHD-related health needs and helps fill an important gap in the current literature. The findings broaden the scope of health management for children with ADHD beyond traditional neurobehavioral and educational domains, and provide foundational data to inform the development of targeted oral health interventions for this underserved population.

Several limitations of this study should be acknowledged. First, all participants were recruited from a single maternal and child health hospital in Dezhou, Shandong Province, which may limit the generalizability of our results to populations receiving care under different medical systems or regional contexts. Second, the case-control design cannot establish causal links between ADHD diagnosis and adverse oral health outcomes, only statistical associations. Third, the sample of children with ADHD (*n* = 130) was relatively modest, limiting statistical power to identify weaker associations and preventing analyses stratified by ADHD subtype, medication category, or dosage levels. Fourth, oral hygiene behaviors and dietary habits were assessed by parental self-report and may therefore be subject to recall and social desirability bias. Fifth, caries screening was conducted solely via clinical visual and tactile examination without dental radiography, likely leading to undercounting of interproximal caries lesions. Sixth, information on ADHD symptom severity and exact medication dosage were unavailable, making it impossible to analyze dose–response relationships between treatment and oral disease risk. Given these constraints, the effect sizes reported in the multivariate regression models ought to be interpreted cautiously. In particular, the wide confidence intervals around several adjusted odds ratios reflect sparse subgroup cell counts and quasi-complete separation, precluding definitive conclusions about the exact magnitude of observed associations. The absolute aOR values should therefore not be overinterpreted, and the findings should be viewed as hypothesis-generating rather than confirmatory. Subsequent larger-scale, multi-centre studies are needed to replicate our findings and mitigate statistical instability caused by sparse subgroup cell counts.

To address these limitations, future studies should adopt prospective cohort designs with larger, multi-center samples, and incorporate radiographic imaging to improve the comprehensiveness of oral disease diagnosis. Detailed data on ADHD subtypes, medication types, and dosages should also be collected. Larger sample sizes are particularly critical to minimize sparse cell counts and quasi-complete separation, thereby reducing the risk of unstable effect estimates and excessively wide confidence intervals; penalized regression methods may still be reserved as a supplementary sensitivity check even in adequately powered cohorts. In addition, objective measures of oral hygiene behaviors, such as direct observation or digital monitoring, should also be integrated with self-reported questionnaires to reduce measurement bias. Such studies will build a more robust evidence base, generate targeted intervention guidance for children with ADHD, translate research outcomes into clinical practice, and ultimately improve oral health outcomes for this vulnerable group.

## Conclusions

Children with ADHD in Dezhou City had notably poorer oral health compared with healthy peers, presenting with a higher prevalence of dental caries, lower rates of dental restoration, and compromised periodontal conditions. These differences were associated with inadequate oral hygiene practices, frequent consumption of high-sugar foods, and suboptimal oral health self-management behaviors, which were closely related to ADHD-related inattention and impulsivity. In addition, limited parental supervision and lower socioeconomic status were also associated with increased oral health risks. These findings highlight the need for a comprehensive management approach integrating preventive dental care, targeted clinical treatment, tailored behavioral interventions, family support, and health equity initiatives to improve oral health among children with ADHD.

## Supplementary Information


Supplementary Material 1.



Supplementary Material 2.



Supplementary Material 3.



Supplementary Material 4.


## Data Availability

The datasets generated and/or analysed during the current study are available from the corresponding author on reasonable request.

## Abbreviations

ADHD: Attention-Deficit/Hyperactivity Disorder
aOR: Adjusted Odds Ratio
CI: Confidence Interval
DMFT: Decayed, Missing, Filled Teeth (permanent dentition)
dmft: Decayed, missing, and filled teeth (primary dentition)
MGI: Modified Gingival Index
PI: Plaque Index
SD: Standard Deviation
