# Supplementary material for: Oral health status and associated influencing factors in children with attention-deficit/hyperactivity disorder: a case-control study
Source: BMC Oral Health. 2026 Aug 19;26:1629. doi: 10.1186/s12903-026-09605-8 (PMC13526303; doi:10.1186/s12903-026-09605-8)
Supplement: Supplementary file 1 [file 12903_2026_9605_MOESM3_ESM.pdf]

Participant Flow Diagram

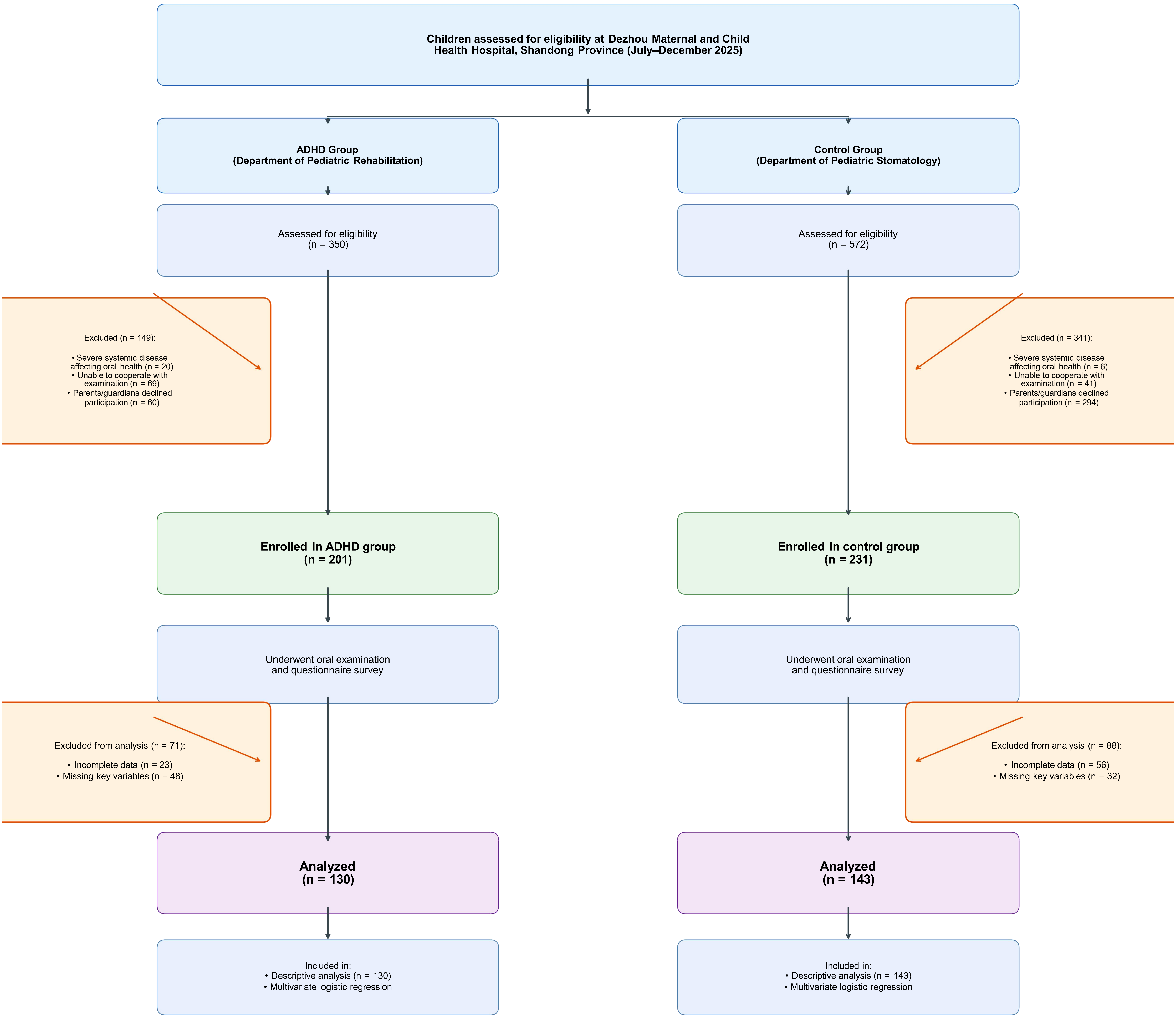

Screening exclusion criteria:
(1) Severe systemic disease affecting oral health; (2) Inability to cooperate with oral examination; (3) Parents or guardians declined to participate.
Post-enrollment exclusion: Incomplete questionnaire data or missing key variables identified after oral examination and survey completion.

ADHD = Attention-Deficit/Hyperactivity Disorder; DSM-5 = Diagnostic and Statistical Manual of Mental Disorders, 5th Edition.
