# Supplementary material for: Oral health status and associated influencing factors in children with attention-deficit/hyperactivity disorder: a case-control study
Source: BMC Oral Health. 2026 Aug 19;26:1629. doi: 10.1186/s12903-026-09605-8 (PMC13526303; doi:10.1186/s12903-026-09605-8)
Supplement: Supplementary file 2 [file 12903_2026_9605_MOESM1_ESM.docx]

**Supplementary File 1**

# Oral Health Questionnaire

（多动症儿童口腔健康调查问卷）

Dear participants, thank you for participating in this health education questionnaire survey. This survey aims to provide targeted health intervention services and improve the quality of health education in our department. Please answer based on your knowledge. Thank you for your cooperation!

**Child's Name:** ______________ **Parent's Name:** ______________ **Phone:** ______________

## 1. Basic Information

1. Child's Date of Birth: [ Year Month Day ] Sex: □ Male □ Female

2. Who primarily takes care of the child in daily life?

□ Father □ Mother □ Grandparents □ Others: ________

3. At what age was the child diagnosed with ADHD?

________ years old

4. Does the child have a long-term medication history (e.g., Methylphenidate)?

□ Yes (Medication name: ________) □ No

## 2. Oral Health Status

5. Caries status (Clinical examination data may be combined; parents report subjective perception):

5.1 Have you visited a dentist for caries in the past year? □ Yes □ No

5.2 Does the child currently have untreated dental caries? □ Yes (Number: ____) □ No

6. Gum health: Does the child's gums often bleed during tooth brushing?

□ Daily □ A few times a week □ Occasionally □ Never

7. Oral treatment needs: What treatments does the child need? (Multiple choice)

□ Fillings □ Extraction □ Orthodontics □ Periodontal treatment □ None

## 3. Daily Behavioral Habits

**8. Brushing habits:**

8.1 At what age did the child start brushing teeth?

□ Before age 1 □ Age 2-3 □ Age 3-4 □ Age 4-5 □ Age 5-6 □ Has not started

8.2 How many times does the child brush teeth per day?

□ More than 2 times □ Once □ Often forgets □ Has not started

8.3 Average brushing time: □ Less than 1 min □ 1-2 min □ 2-3 min □ More than 3 min

8.4 Supervision: Do you supervise the child while brushing?

□ Daily □ Weekly □ Sometimes □ Occasionally □ Never

9. Dietary habits: Frequency of the following foods consumed by the child per week

| **Food Type** | **Almost Daily** | **3-4 Times/Week** | **Occasionally** | **Never** |
| --- | --- | --- | --- | --- |
| Candy/Chocolate | □ | □ | □ | □ |
| Carbonated Drinks | □ | □ | □ | □ |
| Sticky Snacks (e.g., gummies) | □ | □ | □ | □ |

## 4. ADHD-Related Behavioral Impact

**10. Behavioral manifestations (Check based on actual situation):**

Does the child experience the following problems due to hyperactivity/inattention?

| **Problem** | **Often** | **Sometimes** | **Rarely** | **Never** |
| --- | --- | --- | --- | --- |
| Forgets to brush teeth | □ | □ | □ | □ |
| Insufficient brushing time or perfunctory brushing | □ | □ | □ | □ |
| Resists parental supervision during brushing | □ | □ | □ | □ |
| Impulsively eats high-sugar snacks | □ | □ | □ | □ |

11. Does the child have difficulty cooperating with dental treatment due to ADHD symptoms?

□ Very difficult □ Quite difficult □ Average □ Relatively easy □ No difficulty

## 5. Parent-Related Factors

**12. Parent's knowledge level: Are you aware of the following? (Multiple choice)**

□ Fluoride toothpaste prevents cavities

□ How to use dental floss/interdental brushes

□ Importance of regular dental check-ups

□ Relationship between high-sugar diet and dental caries

□ None of the above

**13. Family support (For medical resource analysis only):**

13.1 Family monthly income range:

□ <3000 CNY □ 3000-6000 CNY □ 6000-10000 CNY □ >10000 CNY

13.2 Highest parental education level:

□ Primary school or below □ Middle/High school □ College □ Master's degree or above

## 6. Oral Bad Habits

14. Are there any oral bad habits? □ No

□ Yes: One-sided chewing (Duration: ________)

Thumb/dummy sucking (Duration: ________)

Tongue thrust/Lower lip licking/Upper-lower teeth contact (Duration: ________)

Object/finger biting (Duration: ________)

Mouth breathing (Duration: ________)

Chin support (Duration: ________)

## 7. Oral Health Service Utilization

15. When was the child's last dental visit?

□ Within 6 months □ 6-12 months □ More than 12 months □ Never visited

Reason for recent dental visit:

□ Consultation □ Treatment □ Prevention □ Don't know

*Thank you for completing this questionnaire!*
