# Supplementary material for: Oral health status and associated influencing factors in children with attention-deficit/hyperactivity disorder: a case-control study
Source: BMC Oral Health. 2026 Aug 19;26:1629. doi: 10.1186/s12903-026-09605-8 (PMC13526303; doi:10.1186/s12903-026-09605-8)
Supplement: Supplementary file 4 [file 12903_2026_9605_MOESM4_ESM.docx]

**Firth Penalized Logistic Regression Analysis of Dental Outcomes in Children with ADHD**

Report generated: August 3, 2026 | Outcome variables: Dental Caries, PI ≥ 2, MGI ≥ 2 | Sample: ADHD group (n = 130)

# Abstract

Background: Multivariate logistic regression for dental caries in children with attention-deficit/hyperactivity disorder (ADHD) showed extremely wide confidence intervals (CI) indicative of quasi-complete separation. For plaque index (PI) and gingival index (MGI) models, standard estimates appeared stable but have not been formally verified.

Objective: To apply Firth penalized logistic regression with profile likelihood (PL) CIs across all three dental outcome models, and to compare results with standard maximum-likelihood (ML) estimates to assess robustness.

Methods: Firth penalized logistic regression was fitted to data from 130 children with ADHD, with profile likelihood confidence intervals (PL CIs) derived via inversion of the penalized likelihood ratio statistic. Variable sets entered into each Firth model strictly matched the covariate lists of the corresponding primary logistic regression models: the full pre-specified covariate set from forced-entry regression for dental caries, and the reduced covariate sets identified via backward stepwise likelihood ratio (LR) selection in SPSS for PI and MGI outcomes, respectively.

Results: For caries, Firth estimates were substantially more conservative than standard ML, confirming quasi-complete separation. For PI and MGI, Firth and standard ML estimates were highly consistent, supporting the robustness of those findings.

Conclusion: All three outcome models yield directionally consistent and statistically significant associations under Firth penalization. Profile likelihood CIs are preferred over Wald CIs in sparse data settings (Heinze & Schemper, 2002).

# Methods

Study population. Data were obtained from 130 children diagnosed with ADHD. Three dental outcomes were analysed: (1) presence of dental caries (yes/no), (2) PI ≥ 2 (poor oral hygiene, yes/no), and (3) MGI ≥ 2 (gingivitis, yes/no).

Distinct covariate selection strategies were implemented across the three study outcomes. Primary caries model: All five pre-specified clinically relevant covariates (gender, primary caregiver, candy consumption frequency, parental education level, and methylphenidate medication use) were retained via forced entry, without interaction terms, consistent with the primary multivariate analysis reported in the main manuscript. Primary plaque index (PI) and modified gingival index (MGI) models: Final covariate sets were identified using backward stepwise likelihood ratio (LR) selection in SPSS, with a removal threshold set at P > 0.05. For sensitivity testing via Firth penalized logistic regression, we applied the exact same finalized covariate sets obtained from each corresponding primary model to their respective Firth models: the full forced-entry covariate list for caries, and the backward LR-reduced covariate lists for PI and MGI.

Firth penalized regression. To address quasi-complete separation and small-sample bias, Firth (1993) penalized logistic regression was applied. The penalized log-likelihood is L*(β) = L(β) + ½ log|I(β)|, where I(β) is the Fisher information matrix. This approach yields finite estimates even under complete or quasi-complete separation.

Confidence intervals. Profile likelihood (PL) CIs were computed by inverting the penalized likelihood ratio test. PL CIs have superior coverage properties compared with Wald (normal-approximation) CIs in sparse data and are the recommended approach for Firth regression (Heinze & Schemper, 2002; Firth, 1993).

Statistical software. Firth regression and PL CIs were implemented in Python using custom penalized likelihood optimization (scipy.optimize). Standard logistic regression models were fitted in SPSS v26 with distinct variable selection approaches by outcome: forced-entry regression for dental caries, and backward stepwise likelihood ratio (LR) selection for PI and MGI periodontal outcomes.

## Table 1. Variable Definitions

| **Variable (English)** | **Original Name** | **Coding** | **Model(s)** |
| --- | --- | --- | --- |
| Candy consumption | 吃甜食二分类 | 0 = Low, 1 = High | Caries |
| Medication use | 服药情况二分类 | 0 = No, 1 = Yes | Caries |
| Higher parental edu. | 最高学历二分类 | 0 = ≤High school, 1 = College+ | Caries |
| Gender (female) | 性别 | 0 = Male, 1 = Female | Caries |
| Primary caregiver (parents) | 主要照护者 | 0 = Others, 1 = Parents | Caries |
| Brushing neglect | 刷牙敷衍二分类 | 0 = Adequate, 1 = Neglect | PI |
| High income | 收入二分类 | 0 = Low, 1 = High | PI, MGI |
| Brushing ≥2/day | 每天刷牙次数二分类 | 0 = <2/day, 1 = ≥2/day | PI |
| Forgetting to brush | 忘记刷牙二分类 | 0 = No, 1 = Yes | MGI |
| Parental supervision | 监督情况二分类 | 0 = No, 1 = Yes | MGI |
| Disease duration >3 yr | 患病时间二分类 | 0 = ≤3 yr, 1 = >3 yr | MGI |

# Results

## Descriptive Statistics

Among 130 children with ADHD, 105 (80.8%) had dental caries, 91 (70.0%) had PI ≥ 2, and 79 (60.8%) had MGI ≥ 2.

## Model Results

**Table 2. Dental Caries — Primary Multivariate Logistic Regression vs Firth Penalized LR (n = 130)**

| **Variable** | **Standard LR OR (95% Wald CI)** | **P** | **Firth OR (95% Profile LR CI)** | **P** | **Consistency** |
| --- | --- | --- | --- | --- | --- |
| Candy consumption, high (Ref: Low) | 84.54 (12.2–584) | < 0.0001 | 37.27 (8.50–237.75) | < 0.0001 | Yes |
| Methylphenidate use, yes (Ref: No) | 39.65 (15.79–271) | < 0.0001 | 22.13 (5.12–132.10) | < 0.0001 | Yes |
| Higher parental education (Ref: ≤High school) | 0.10 (0.014–0.684) | 0.019 | 0.153 (0.022–0.738) | < 0.0001 | Yes |
| Gender, female (Ref: Male) | 0.32 (0.059–1.77) | 0.194 | 0.360 (0.069–1.675) | 0.194 | Yes |
| Primary caregiver, parents (Ref: Others) | 3.35 (0.262–42.7) | 0.352 | 1.859 (0.084–27.96) | 0.352 | Yes |

Interpretation: The primary multivariate logistic regression model (forced-entry, all five pre-specified covariates) yielded extremely wide Wald CIs for candy consumption (aOR = 84.54, 95% CI: 12.2–584) and methylphenidate use (aOR = 39.65, 95% CI: 15.79–271), consistent with quasi-complete separation. Firth penalized estimates are substantially more conservative and reliable: the OR for candy consumption was attenuated from 84.54 (standard ML) to 37.27 (Firth), and the OR for methylphenidate use from 39.65 to 22.13. Parental education level showed a protective association (aOR = 0.10, 95% CI: 0.014–0.684; Firth OR = 0.153, 95% PL CI: 0.022–0.738, P < 0.0001). Gender (aOR = 0.32, 95% CI: 0.059–1.77) and primary caregiver (aOR = 3.35, 95% CI: 0.262–42.7) were not significantly associated with dental caries in either the standard or penalized model. All three significant variables retained their direction and significance under Firth penalization, supporting the robustness of the findings despite sparse data.

**Table 3. PI ≥ 2 (Plaque Index) — Standard LR vs Firth Penalized LR (n = 130)**

| **Variable** | **Standard LR OR (95% Wald CI)** | **P** | **Firth OR (95% Profile LR CI)** | **P** | **Consistency** |
| --- | --- | --- | --- | --- | --- |
| Brushing neglect (Yes vs No) | 8.782 (3.120–24.71) | <0.001 | **8.082 (3.045–23.08)** | < 0.001 | Yes |
| High income (Yes vs No) | 0.293 (0.116–0.739) | 0.009 | **0.308 (0.122–0.748)** | 0.009 | Yes |
| Brushing ≥2/day (Yes vs No) | 0.142 (0.047–0.427) | 0.001 | **0.153 (0.050–0.435)** | < 0.001 | Yes |

Interpretation: Standard and Firth estimates are highly consistent for PI. Brushing neglect was the strongest risk factor; high income and higher brushing frequency were independently protective. No evidence of separation issues in this model.

**Table 4. MGI ≥ 2 (Gingival Index) — Standard LR vs Firth Penalized LR (n = 130)**

| **Variable** | **Standard LR OR (95% Wald CI)** | **P** | **Firth OR (95% Profile LR CI)** | **P** | **Consistency** |
| --- | --- | --- | --- | --- | --- |
| Forgetting to brush (Yes vs No) | 2.887 (0.955–8.725) | 0.060 | **2.711 (0.946–8.174)** | 0.063 | Yes |
| Parental supervision (Yes vs No) | 0.111 (0.040–0.307) | <0.001 | **0.126 (0.045–0.319)** | < 0.001 | Yes |
| High income (Yes vs No) | 0.328 (0.129–0.835) | 0.019 | **0.349 (0.138–0.850)** | 0.020 | Yes |
| Disease duration >3 yr (Yes vs No) | 11.30 (3.782–33.75) | <0.001 | **9.696 (3.649–30.12)** | < 0.001 | Yes |

Interpretation: Parental supervision and disease duration >3 years were the strongest predictors of MGI ≥ 2. Forgetting to brush showed borderline significance (P = 0.060 by standard LR); Firth estimates for this variable should be interpreted cautiously. High income remained independently protective.

*Figure S1. Forest plot of Firth penalized logistic regression for dental caries (DMFT+dmft > 0) in children with ADHD (n = 130). ORs with 95% profile likelihood confidence intervals are shown.*

# Discussion

This sensitivity analysis demonstrates that Firth penalized logistic regression provides more reliable estimates than standard ML in the presence of quasi-complete separation (dental caries model). The penalized ORs for candy consumption (37.27) and methylphenidate use (22.13) are markedly attenuated compared with the primary standard ML estimates (84.54 and 39.65, respectively), confirming that the primary analysis was affected by quasi-complete separation. Nevertheless, both associations remain highly statistically significant (P < 0.0001). For PI and MGI models where separation was not a concern, Firth and standard estimates converged, confirming the robustness of the reported associations.

The use of profile likelihood CIs — rather than Wald (normal-approximation) CIs — is strongly recommended for Firth regression. Wald CIs rely on quadratic approximation of the likelihood and have poor coverage rates under sparse data conditions. Profile LR CIs are obtained by direct inversion of the likelihood ratio statistic and maintain near-nominal coverage rates (Heinze & Schemper, 2002).

Limitations: The wide confidence intervals for candy consumption and methylphenidate use reflect genuine data sparsity in certain covariate combinations. Firth regression provides the best achievable finite estimates under these conditions, but the exact magnitude of these associations should be interpreted with caution. The primary focus should be on the direction and statistical significance of the associations. Future studies with larger samples are warranted.

# References

Firth D. (1993). Bias reduction of maximum likelihood estimates. Biometrika, 80(1), 27–38.

Heinze G, Schemper M. (2002). A solution to the problem of separation in logistic regression. Statistics in Medicine, 21(16), 2409–2419.

Heinze G, Ploner M. (2004). A SAS macro, S-PLUS library and R package to perform logistic regression without convergence problems. Technical Report 2/2004, Section of Clinical Biometrics, Medical University Vienna.

Venzon DJ, Moolgavkar SH. (1988). A method for computing profile-likelihood-based confidence intervals. Applied Statistics, 37(1), 87–94.
